# Dual inhibition of BET and EP300 has antitumor activity in undifferentiated pleomorphic sarcomas and synergizes with ferroptosis induction

**DOI:** 10.1016/j.tranon.2024.102236

**Published:** 2024-12-15

**Authors:** Stéphanie Verbeke, Aurélien Bourdon, Mathilde Lafon, Vanessa Chaire, Bertolo Frederic, Amina Naït Eldjoudi, Marie-Alix Derieppe, Francis Giles, Antoine Italiano

**Affiliations:** a Sarcoma Unit, Bergonié Institute 33000 Bordeaux, France; b INSERM U1312 BRIC BoRdeaux Institute of onCology, University of Bordeaux 33000 Bordeaux, France; c Service Commun des Animaleries, University of Bordeaux 33000 Bordeaux, France; d Epigene Therapeutics, Saint Laurent QC, Canada; e Faculty of Medicine, University of Bordeaux 33000 Bordeaux, France

**Keywords:** BET, Sarcoma, Ferroptosis, CRISPR screen

## Abstract

Undifferentiated pleomorphic sarcoma (UPS) is the most frequent and the most aggressive sarcoma subtype for which therapeutic options are limited. The identification of new therapeutic strategies is therefore an important medical need. Epigenetic modifiers has been extensively investigated in recent years leading to the development of novel therapeutic agents. Dual BET/EP300 inhibitors have shown synergistic antitumor activity and have recently entered clinical development. To date, no data related to potential of BET/EP300 inhibition as a treatment in UPS have been reported. To investigate the therapeutic potential of BET/EP300 inhibition, we evaluated the antitumor activity of three compounds in vitro via MTT, apoptosis and cell cycle assays. The most potent inhibitor was evaluated in vivo in two animal models and the mechanisms of action were investigated by RNA sequencing, Western blotting and immunofluorescence staining. A CRISPR knockout screen was performed to identify resistance mechanisms. Among the three compounds tested, the dual inhibitor NEO2734 was the most potent, decreased the viability of UPS cells in vitro through a regulation of E2F targets and cell cycle and decreased the tumor growth in vivo. Moreover, we identified GPX4 as a gene involved in resistance and showed synergy between BET inhibition and ferroptosis induction.

The present study demonstrated that dual BET/EP300 inhibitors have a relevant antitumor activity in a subgroup of UPS characterized by expression of MYC-targets pathway and identified a potent combination therapeutic strategy that deserves further investigation in the clinical setting.

## Highlights

• A subset of UPS characterized by c-MYC activation may benefit from BET bromodomain inhibition.
• Dual BET/EP300 inhibition shows potent antitumor activity in UPS compared to single BET inhibition.
• The dual BET/EP300 inhibitor NEO2734 decreases viability of UPS cells and reduces tumor growth in vivo.
• GPX4 identified as a key gene in resistance to BET/EP300 inhibition.
• Combination of BET inhibition and ferroptosis induction is highly synergistic.

## Introduction

Soft-tissue sarcomas (STSs) constitute rare, heterogeneous malignancies of connective tissues representing 1 % of cancers in adults and 15 % of cancers in children [1]. The most common of the more than 70 distinct histologies identified are leiomyosarcoma (LMS), liposarcoma (LPS), and undifferentiated pleomorphic sarcoma (UPS). UPS, formerly known as malignant fibrous histiocytoma (MFH), is a high-grade aggressive STS and represents a diagnosis of exclusion based on the absence of a specific line of differentiation after careful histological examination and judicious use of ancillary techniques [2]. Based on a multidimensional analysis, our group recently reported that there are two main disease entities of UPS with distinct immune phenotypes, prognoses and molecular features [3]. One type is significantly enriched in immune response and metabolism (glycolysis and oxidative phosphorylation) pathways, whereas the other type is mainly enriched in MYC targets and epithelial mesenchymal transition (EMT) pathways and is characterized by worse outcome. Despite the heterogeneity among UPS cases, a common genetic feature is low mutation burden. Increasing evidence suggests that many STS cells exhibit epigenetic dysregulation, which plays a crucial role in tumorigenesis [4]. Bromodomain and extraterminal domain (BET) proteins, cyclic adenosine monophosphate response element-binding protein (CBP), and the E1A-binding protein of p300 (EP300) are epigenetic modifiers that facilitate gene transcription through interactions with acetylated histones and/or transcription factors. Their dysregulation leads to aberrant transcriptional outputs, such as increased expression of oncogenes [5].

Despite adequate locoregional treatment, up to 40 % of patients with UPS will develop metastatic disease. Doxorubicin represents the standard first line treatment for patients with advanced disease. However, its activity is limited, with a response rate of only 10 % and a progression-free survival time of less than 6 months [6]. The identification of new therapeutic strategies is therefore an important medical need.

Several BET inhibitors have been investigated in early phase studies that enrolled patients with several types of cancers. Unfortunately, these drugs have limited clinical activity when used as single agents because of dose-limiting toxicities (DLTs) precluded escalation to a dose that would be efficient [7]. There are several findings suggesting that the antitumor activity of BET inhibitors may be enhanced by concomitantly inhibiting histone modifiers such as histone acetyltransferase (HAT) [8]. EP300, one of several non-BET, bromodomain-containing proteins, is the most investigated HAT. The EP300 bromodomain plays a crucial role in chromatin remodeling and gene expression regulation. Moreover, the EP300 bromodomain has been shown to be essential for oncogenic cMYC expression and cell proliferation [9]. Interestingly, the combination of EP300 inhibition with BET inhibition has shown synergistic antitumor activity in preclinical models of myeloma, lymphoma, leukemia and prostate cancer [[10], [11], [12], [13]]. This stronger efficacy of dual inhibitor suggest that using lower doses than when these drugs are administered alone may avoid excessive toxicity in humans. Therefore, novel agents that are dual BET/EP300 inhibitors have recently entered clinical development (NCT05488548).

To date, no data related to the biological role of BET/EP300 inhibition or its potential as a treatment in UPS have been reported. Based on the known mechanisms of action of BET/EP300 inhibition in other cancers (i.e. MYC pathways inhibition) and that at least part of the UPS showed an enrichment of MYC targets, we hypothesized that targeting BET/EP300 proteins in UPS might be an interesting strategy. We therefore aimed to investigate the antitumoral effects of BET/EP300 inhibition in UPS and the related mechanisms of action.

## Materials and methods

### Cell culture and drugs

The undifferentiated pleomorphic sarcoma (UPS) cell lines used in this study called IB106, IB119, JR588 and KN473 were derived from human UPS surgical specimens after obtaining written, informed patient consent and Bergonié Institute Institutional Review Board approval. Each home-made cell line was established as previously described [14] and characterized by array comparative genomic hybridization every 10 passages until p50 to verify that its genomic profile was still representative of the originating tumor sample. No drift in the cell line maintenance or genetic imbalances were shown along passages. Cells were grown in RPMI medium 1640 GlutaMAX^TM^ Supplement (Life Technologies, Carlsbad USA) in the presence of 10 % (v/v) fetal bovine serum and Penicillin/Streptomycin 1 % (Thermo Scientific, Gibco^TM^), in flasks. Cells were maintained at 37 °C in a humidified atmosphere containing 5 % CO2. Cells were routinely passaged every 3 days and all the experiments were performed with cell lines between passages 25 and 60.

CPI-637, is a potent dual inhibitor of CBP/EP300. NEO1132 and NEO2734 are chemically distinct dual inhibitors of both BET and CBP/EP300 proteins with different affinity for their targets. The three compounds were provided by Epigene Therapeutics Inc. JQ1 is a pan-BET inhibitor purchased from MedChemExpress. All the compounds were prepared as a 10 mM stock solution in DMSO and stored at -20 °C for in vitro studies or prepared weekly as 10 mg/kg doses in 40 % polyethylene glycol (PEG400) in distilled water (vehicle) for in vivo studies (NEO2734). To induce ferroptosis, RSL3, a pharmacological inhibitor of GPX4 was purchased from MedChemExpress (Monmouth Junction, New Jersey, USA), prepared as a 10 mM stock solution in DMSO and stored at -80 °C for in vitro studies.

### Cell viability assay and synergy

UPS cells were seeded in triplicate at 4000 cells/well into 96-well plates, cultured with fresh growth medium for at least 24 h and treated with a range of increasing concentrations of drugs (BET inhibitors) for 72h (0.0001 / 0.001 / 0.01 / 0.1 / 1 / 5 /10 / 50 / 100 / 200 µM). Cell viability was assessed by MTT (2-deoxyglucose (2-DG) and 3–4,5-dimethylthiazol-2-yl)-2,5-diphenyltetrazolium bromide) assay (Sigma-Aldrich, St. Quentin Fallavier, France) at a final concentration of 0.5 mg/mL and 3 h of incubation. Then, supernatant was discarded, 100 µL of dimethyl sulfoxide (DMSO) was added and the absorbance at 570 nm was monitored using a Flexstation 3 Plate reader (Molecular Devices, Sunnyvale, USA), using 630 nm as a reference. The half-maximal inhibitory concentration (IC50) was calculated with GraphPad Prism software version 6.0 for Windows (GraphPad Software, RRID: SCR_002798, La Jolla, USA). Each experiment was repeated at least 3 times.

For RSL3 and NEO2734 used in combination, cells were treated with a range of increasing concentrations of RSL3 and NEO2734 for 72 h After MTT assay and absorbance measurement, the synergy was determined using SynergyFinder 3.0, a web application for interactive analysis and visualization of multi-drug combination response data (http://www.synergyfinder.org/) [15]. The synergy score was quantified using the Bliss reference model. The score is averaged over all the dose combination measurements, giving a positive or negative value, corresponding to synergism (red) or antagonism (green), respectively. The most synergistic area (MSA) score represents the most synergistic 3-by-3 dose-window in a dose-response matrix.

### Cell cycle analysis and apoptosis assay

UPS cells (5 × 10^4^ cells/well) were seeded in 6-well plates in triplicate. For apoptosis assessment, 24 h after seeding, cells were treated with drugs at the IC50 or at 25 µM for IB119 and KN473 for 48 h Before flow cytometry analysis (FACS Calibur flow cytometer, BD biosciences, San Jose, CA, USA), cells were washed once with phosphate-buffered saline (PBS) and labeled with annexin-V-FITC and propidium iodide (PI) according to the manufacturer's protocol (BD Biosciences, San Jose, CA, USA). The percentage of cells in early apoptosis (annexin-V-positive, PI-negative) and in late apoptosis or necrosis (annexin-V and PI-positive) was calculated using FlowJo version 7.6.3 (RRID: SCR_008520). The percentages of overall death (sum of early and late apoptosis) are represented as the mean ± SEM values based on 3 independent experiments.

For cell cycle analysis, cells were synchronized in serum-free medium overnight and then treated for 24 h with drugs at the IC50 or at 25 µM for IB119 and KN473.Cells were then permeabilized with 70 % ethanol at -20 °C overnight. Following ethanol removal, cells were washed with PBS and stained with a Propidium Iodide (PI) and ribonuclease-containing solution before measurement of DNA content by FACS. The data were analyzed with FlowJo software and the results were expressed in terms of percentage of cells in a given phase of the cell cycle based on 3 independent experiments.

### Western blot analysis

Cells were treated with or without BET inhibitors at IC50 for 72 h Cells were harvested in 100 µl of radio-immuno-precipitation assay (RIPA) lysis buffer. The lysate was centrifuged (13 000 rpm, 15 min, 4 °C), and the supernatant was stored at -20 °C. Equal amounts of total protein (30 µg) were electrophoresed on 12 % or 8 % sodium dodecyl sulfate polyacrylamide gels and transferred onto polyvinylidene difluoride membranes. The blots were probed overnight at 4 °C with an anti-c-MYC (Cell Signaling Technology Cat# 5605, RRID:AB_1,903,938), anti-actin (Sigma-Aldrich Cat# A3853, RRID:AB_262,137), anti- p21 (Cell Signaling Technology Cat# 2947, RRID:AB_823,586), anti-GAPDH (Santa Cruz Biotechnology Cat# sc-51,907, RRID:AB_629,537), anti-CDK1 (Cell Signaling Technology Cat# 9116, RRID:AB_2,074,795), anti-CDK2 (Cell Signaling Technology Cat# 2546, RRID:AB_2,276,129), anti-PLK1 (Cell Signaling Technology Cat# 4513, RRID:AB_2,167,409), anti-AURKA (Cell Signaling Technology Cat# 14,475, RRID:AB_2,665,504), anti-cyclin B1 (Thermo Fisher Scientific Cat# MA1–155, RRID:AB_2,536,863), anti-GPX4 (Cell Signaling Technology Cat# 52,455, RRID:AB_2,924,984) primary antibody diluted in PBST (DPBS 10X (GibcoTM) after 1X dilution; 0.1 % Tween-20) with 5 % bovine serum albumin. The horseradish peroxidase-conjugated secondary antibody anti-mouse (GE Healthcare Cat# NA931, RRID:AB_772,210) or anti-rabbit (GE Healthcare Cat# NA934, RRID:AB_772,206) was diluted at 1:5000. Bound antibodies were visualized on Fusion Fx7 imaging system (Fisher Bioblock Scientific, Waltham, USA) using the Immobilon^TM^ Western enhanced chemiluminescence detection kit (Millipore Corporation, Billerica, USA). The resulting bands were analyzed and quantified using ImageJ 1.48v software (RRID:SCR_003070, National Institutes of Health, Bethesda, USA).

### Animal study

All animal experiments were performed with the approval of the institutional animal use and care committee under project license APAFiS #20,350- 2,019,041,812,178,984. This study followed the French and European Union guidelines for animal experimentation (RD 1201/05, RD 53/2013 and 86/609/CEE, respectively). PDX JR588 or PDX KN473 were implanted subcutaneously into the right flank of 6- to 8-week-old Rag2-/- γc-/- mice which were procured and housed in the institutional animal facilities. Once palpable, tumor volumes were calculated using the following formula: length × width^2^/2. Seventeen days (PDX JR588) or 46 days (PDX KN473) after implantation when the average size of the tumors was around 100 mm^3^, mice were randomized into 2 groups (n = 8 and n = 6 mice per group for JR588 and KN473 respectively) and treated orally by vehicle (40 % PEG400) or NEO2734 at 10 mg/kg QD (5 days-on/2 days-off). Tumor size was measured every 2–3 days with calipers until a limit size of 2000 mm^3^ and then mice were euthanized. Tumor progression and mice body weight were analyzed with GraphPad Prism software using two-way ANOVA test and Bonferoni post-hoc test.

### Reactive oxygen species assessment

Cells were seeded in 6-well plates (200,000 cells/well for IB106 and JR588, 150,000 cells/well for IB119 and KN473). After 24 h, cells were treated with 20 µM of DCFDA (DCFDA/H2DCFDA cellular ROS Assay Kit, ab113851, Abcam) in serum free medium (500 µL/well) and incubated in dark at 37 °C during 30 min. Cells were then washed with PBS and treated with RSL3 and/or NEO2734 during 24 h After the treatment, floating and attached cells were collected by centrifugation, resuspended in 300 µL of PBS and analyzed by flow cytometry. Percentage of ROS-positive cells was calculated using FlowJo v10.8.1 software.

### Lipid peroxidation measurement

To assess the peroxidized lipids, we used the BODIPY^TM^ 581/591 C11 sensor (Invitrogen D3861, ThermoFisher), an undecanoic acid used to detect ROS in cell membranes. The oxidation of polyunsaturated butadienyl portion of the dye results in shift of fluorescence emission peak from 590 nm to 510 nm measured by flow cytometry. As for ROS assessment, cells were seeded in 6-well plates and treated with RSL3 and/or NEO2734 during 24 h After the treatment, floating and attached cells were collected by centrifugation, washed with PBS and resuspended in 800 µL of a PBS solution containing 2.5 µM of BODIPY-C11. The cells were then incubated in the dark during 20 min at 37 °C, centrifuged and resuspended in 400 µL of PBS before FACS analysis. Percentage of BODIPY-positive cells was calculated using FlowJo v10.8.1 software.

### Pooled genome-wide CRISPR screen

Genome-scale CRISPR knock-out (GeCKO) v2.0 pooled libraries (Two vector system) were purchased from Addgene. GeCKO libraries A and B were amplified and prepared as previously described [16,17]. KN473 cells were infected with lentiviral particles of lenti-Cas9 at a multiplicity of infection (MOI) of ∼0.7 followed by selection with blasticidin at 2 µg/ml for 10 days to obtain the stable cell line expressing Cas9. Four hundred-millions of stable cells were then infected with the amplified lentiviral libraries A and B at the MOI of ∼0.3 and seeded in T175 mm flasks with 5  ×  10^6^ cells per flask. Puromycin at 1 µg/ml was added the second day after lentiviral libraries infection and kept for 5 days. After selection, the surviving cells (150.10^6^) were pooled together and split into two groups: 75.10^6^ cells were centrifuged and the pellet was frozen at -20 °C (CONTROL group) and 75.10^6^ cells were treated with NEO2734 (NEO group) for 72 h at 1 µM, a dose that normally doesn't kill the parental KN473 cells. After the treatment, the cells were centrifuged and the pellet was frozen at -20 °C. Frozen cell pellets (CONTROL and NEO group) were thawed and genomic DNA was extracted with a Quick-gDNA MidiPrep kit (Zymo Research) according to the manufacturer's protocol. PCR was performed to prepare sequencing library with the NEBNext HF 2X PCR master mix as described before [18]. The library was sequenced on a MiSeq using the 2 × 150 bp paired-end sequencing protocol (Illumina). To strengthen the results, data analysis was performed by three different methods: the MAGeCK tool [19], the CRISPRCloud2 pipeline (https://crispr.nrihub.org) and the RIGER (RNAi gene enrichment ranking) method launched through GENE-E from the Broad Institute (https://software.broadinstitute.org/GENE-E/extensions.html). RIGER settings were adjusted as described previously [18].

### Lentiviral particle production

Viral particles were produced by transient transfection of specific plasmids (GeCKO library A+B, lentiCas9-Blast, pLentiGuide-Puro GPX4 sgRNA sequence:GAATTTGACGTTGTAGCCCG (GenScript sc1678)) with the packaging plasmids pVSVg (AddGene 8454) and psPAX2 (AddGene 12,260) into HEK293T cells using calcium phosphate transfection. Media was collected 48 h after transfection and centrifuged at 2500 rpm for 3 min to pellet cell debris. The supernatant was filtered through a 0.45 µm PVDF membrane and aliquots were stored at -150  °C.

### RNA sequencing

For RNA sequencing, the four UPS cells were treated or not with NEO2734 at their IC50 or at 25 µM for IB119 and KN473 for 72 h RNA were then extracted using RNeasy Plus Mini Kit (Qiagen, Hilden, Germany) according to the supplier's instructions. The purity and concentration of isolated RNA were determined by spectrophotometer NanoDrop^TM^ and capillary electrophoresis using Bioanalyser 2100 (Agilent Technologies Inc.,Santa Clara, USA) was carried out to determine the RNA integrity number (RIN). RNA-sequencing was performed by Integragen, Inc (Evry, France) on a NovaSeq6000 S2 platform (Illumina Inc. San Diego, CA, USA) via the NEBNext® Ultra™ II mRNA-Seq library kit, paired-end 100 × 2 reads protocol. Number of paired-end reads produced by the sequencer was at least 30M per sample. Bioinformatic analysis was performed as previously described [3]. Briefly, raw RNAseq sequences were controlled for quality using a set of published tools to produce curated reads. Transcript count data were normalized according the VOOM method and the RNAseq differential gene expression between groups of samples was performed using the statistical *t*-test from the R package LIMMA. We defined the significantly up- or down-regulated transcripts using an FDR threshold of 0.05. The fold-change used to further filter the differential gene expression was set to a minimum value of 2. For the gene set enrichment analysis, MSigDB and FGSEA were used to identify pathways or gene ontologies in which the genes of an identified group were enriched.

### Data availability

The data generated in this study have been deposited in NCBI's Gene Expression Omnibus (GEO) and are accessible through GEO Series accession numbers GSE255852 for RNA-seq experiment and GSE255853 for CRISPR screen experiment. Cell lines and PDX availability has to be discussed with the corresponding author and the biological resources center of Bergonié institute.

## Results

### Antitumor activity of BET/EP300 inhibition in UPS

**Fig. 1:**
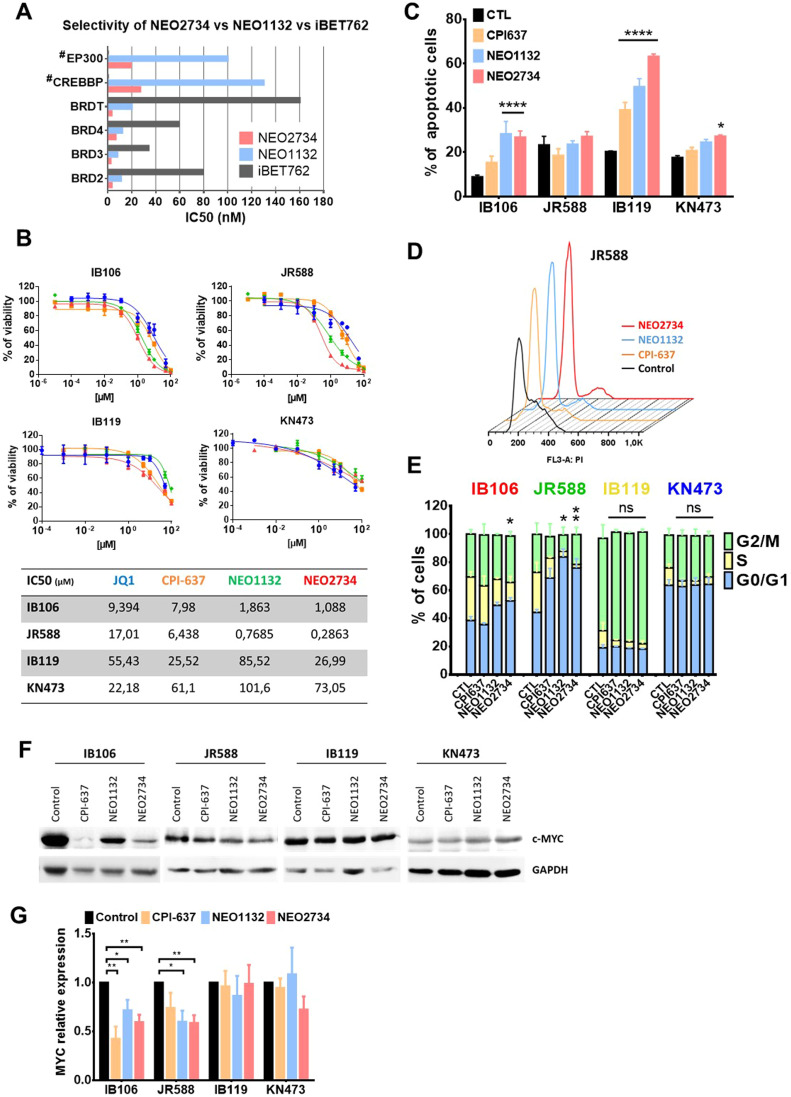
**Anti-tumor activity of BET inhibitors in UPS. A**, Comparison of the binding affinity of NEO2734 with that of NEO1132 and iBET-762 to p300/CBP and a variety of BET bromodomains determined by BROMOscan. Data provided by Epigene Therapeutics. **B**, Assessment of cell viability with BET/CBP/EP300 inhibitors JQ1, CPI-367, NEO1132, NEO2734 in 4 undifferentiated pleomorphic sarcoma (UPS) cell lines by MTT assay. Cells were treated with a range of increasing concentrations of drugs for 72 h and IC50 were calculated with GraphPad Prism software (n = 3 or more). **C**, Apoptosis was assessed by flow cytometry after 48 h of treatment at the IC50 for IB106 and JR588 and at 25 µM for IB119 and KN473. Cells were stained with Annexin-V-FITC and propidium iodide (n = 3, one-way ANOVA, Dunnett's multiple comparisons test). **D and E**, Cell cycle analysis was measured by flow cytometry after propidium iodide staining. After starvation, cells were treated for 24 h at the IC50 for IB106 and JR588 and at 25 µM for IB119 and KN473. Example of cell cycle curve for JR588 in (**D**) (n = 3, two-way ANOVA, Bonferroni's multiple comparisons test). **F**, Western blot of c-MYC in 4 UPS cell lines after 72 h of BET inhibitors treatment at their respective IC50 (or 25 µM for IB119 and KN473). **G**, Quantitation of c-MYC bands in (**F**) using ImageJ software. GAPDH serve as loading control (n = 3; Multiple *t*-tests, Holm-Sidak method).

The most comprehensively characterized BET member is BRD4. Its inhibition impairs the communication between superenhancer (SE) and target promoters, resulting in cell-specific repression of oncogenes. EP300 is also an attractive therapeutic target in cancer due to its roles in promoting growth and transformation. Thus, a novel class of compounds binding both epigenetic readers (BET proteins) and writers (such as EP300) were developed by Epigene Therapeutics. We investigated the antitumor activity in soft-tissue sarcomas of two of these new compounds, NEO1132 and NEO2734. These two structurally unrelated compounds have shown distinct binding affinity in cell-free assay determined by Epigene. NEO2734 binds BRD4 and EP300 with 2–10 fold higher affinity than NEO1132 or i-BET-762, a selective BET inhibitor (Molibresib) (Fig. 1A). We then compared the antitumor activity of these two compounds to inhibitors of CBP/P300 only (CPI-637) and BET proteins only (JQ1) in four UPS cell lines by treatment with increasing concentrations. All four compounds exhibited antitumor activity (Fig. 1B). Overall, the two dual inhibitors were more potent than the monotherapies CPI-637 or JQ1, reinforcing the value of developing this type of molecule. Among the two dual inhibitor, NEO1132 showed lower activity compared to NEO2734 probably due to the distinct affinity for their targets. Moreover, these four UPS cell lines can be separated in two subgroups: IB106 and JR588, were more sensitive, with NEO2734 IC50 values of 1.088 and 0.2863 µM respectively, while IB119 and KN473 cells had IC50 values above 25 µM and 60 µM, respectively with a maximal effect of over 40 % of viable cells remaining in the higher dose. Interestingly, the two sensitive cell lines belong to the immune-low UPS subtype, which is predominantly characterised by MYC target enrichment, epithelial-mesenchymal transition (EMT) pathway. This UPS subtype is associated with a worse clinical outcome, as previously reported [3]. In contrast, the two resistant cell lines were derived from patients belonging to the immune-high subset of UPS. To further describe the mechanism of action of these compounds, we assessed the apoptotic rate and the proliferation rate by cell cycle analysis. In Fig. 1C, we observed a slight induction of apoptosis in IB106 and KN473 cells and a more pronounced induction in IB119 cells, suggesting a different mechanism of action in this cell line. Moreover, we observed G1 phase arrest after BET/EP300 inhibition in the IB106 and JR588 cell lines, and the effect was more pronounced with NEO2734. No significant accumulation was observed in IB119 and KN473 cell (Fig. 1D and E). Since MYC is one of the main targets of BRD4, we examined its protein level after treatment (Fig. 1F and G). In the two sensitive cell lines, IB106 and JR588, we observed a significant decrease in c-MYC expression, whereas in IB119 and KN473 cells, no change was observed, probably explaining their low sensitivity to the compounds. Nevertheless, as apoptosis was observed in IB119 and slightly in KN473 after NEO2734 treatment, mechanisms other than MYC regulation are probably involved in these effects. For example, it has been shown in colorectal cancer (CRC) that NEO2734 treatment can upregulate DR5 or PUMA to induce apoptosis [20]. This may also be due to an aspecific toxic effect, as resistant cell lines (IB119 and KN473) were treated with a high dose of 25 µM.

### Mechanisms of action of BET/EP300 inhibition in UPS

**Fig. 2:**
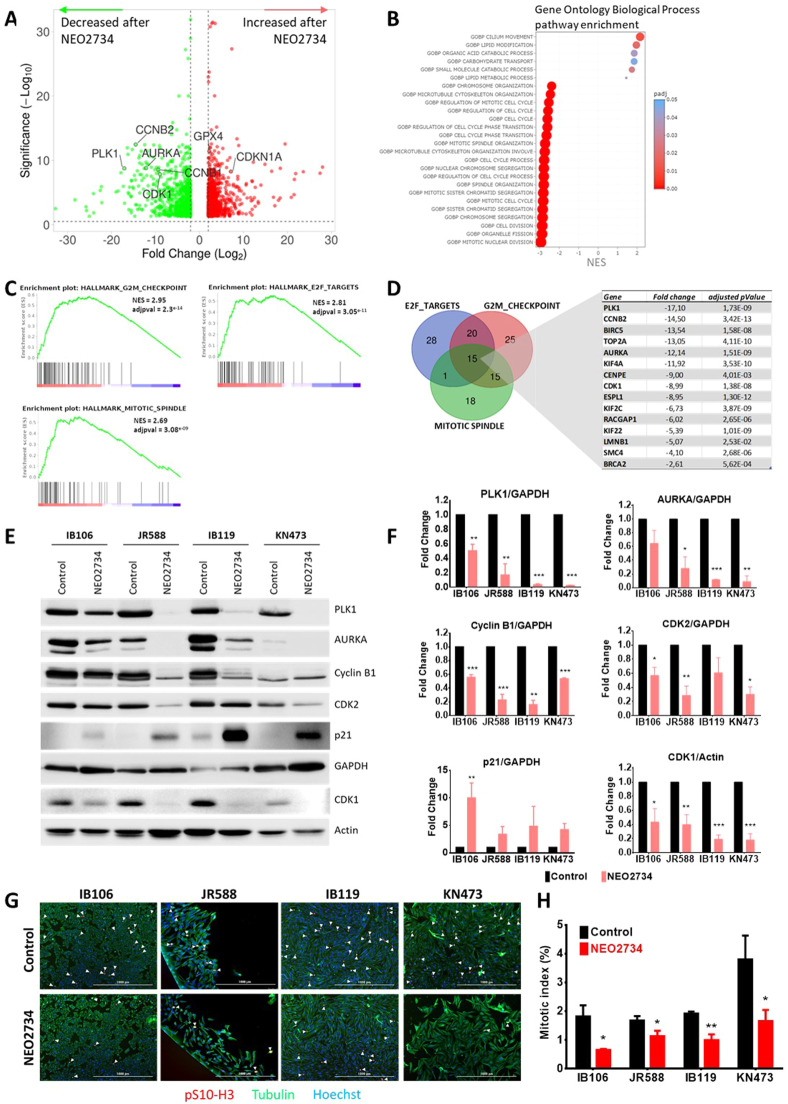
**Dual BET/EP300 inhibitor NEO2734 impact on mitosis. A,** Volcano plot of differential gene expression analysis after RNA sequencing of 4 UPS cell lines treated or not with NEO2734 for 72 h at their respective IC50 (25 µM for IB119 and KN473) in triplicate. 937 genes (green) and 1022 genes (red) were significantly down- or up-regulated respectively after treatment. All cell line data were merged for analysis (12 Control vs 12 NEO-treated cell) and the threshold was –log10 adjusted *p* 〈 0.05 and log2 fold change 〉 2. **B and C,** MSigDB and FGSEA was used to identify Gene Ontologies (GOBP) and Hallmarks pathways enriched in the RNA sequencing experiment. Several biological process involved in mitosis and cell cycle are downregulated after NEO2734 treatment (NES = Normalized Enrichment Score). All cell line data were merged for analysis. **D,** Venn diagram and table of the 15 common genes between the 3 hallmark pathways downregulated. **E,** In vitro validation by Western blot of protein of interest involved in G2/M Checkpoint and identified in the RNA sequencing. Polokinase 1, Aurora Kinase A, Cyclin B1, CDK2 and CDK1 were downregulated after 72 h of NEO2734 treatment in the 4 UPS cell lines whereas p21 was upregulated. **F,** Quantitation of Western blot bands in **(E)** using ImageJ software. GAPDH or Actin serve as loading control (*n* = 3; Multiple *t*-tests, Holm-Sidak method). **G,** Immunofluorescence staining of Tubulin (green) and phosphor-Ser10 Histone H3 (red) as mitosis marker in the 4 UPS cell lines after 24 h of NEO2734 treatment at their respective IC50. Nuclei were stained with Hoechst. White arrows indicate pS10-H3 positive cells. **H,** Mitotic index was calculated by quantitation of pS10-H3 positive cells (cell in mitosis) on total number of cells (Hoechst nuclei stain) with Cytation 3 cell imaging reader. *p < 0.05; **p < 0.01; ***p < 0.001.

To deepen the global mechanisms associated with this antitumoral effect, we analyzed the transcriptomic changes induced by 72 h of treatment with NEO2734 in the four UPS cell lines by RNA sequencing. All cell line data were merged for bioinformatics analysis. We identified 1959 genes that were differentially expressed after dual BET/P300 inhibition (Fig. 2A). Hallmarks and the Gene Ontology gene set from MSigDB were used to identify enriched pathways (Fig. 2B and C). Such analysis revealed a strong transcriptional downregulation of all the processes involved in mitosis (e.g., nuclear division, chromosome segregation, spindle organization) associated with three downregulated hallmark pathways: G2/M checkpoint, mitotic spindle and E2F targets. Fifteen genes were common to these three hallmarks (Fig. 2D). Among them, we confirmed the downregulation at the protein level induced by NEO2734 treatment for Polokinase 1, Aurora Kinase A, Cyclin B1, CDK2 and CDK1, as well as the upregulation of p21 (Fig. 2E and F). These regulated genes are common to all four cell lines as shown in the volcano plot of the individual cell line (supplementary Figure 1 A-D). Interestingly, when we reanalyzed the transcriptomic data by segregating the cell lines into sensitive (IB106 and JR588) and resistant groups (IB119 and KN473) prior to analysis, the results reveal that these key regulatory genes along with the downregulated pathways remained consistently significant in both groups (supplementary Figure 1 E-H). This emphasises the importance of this mechanism of action of dual BET/EP300 inhibitors. Furthermore, the mitotic index was investigated through phosphoS10-Histone H3 staining, which revealed that NEO2734 reduced the number of mitosis in the four UPS cell lines (Fig. 2G and H), thereby confirming its antitumor and cytostatic effects.

**Fig. 3.**
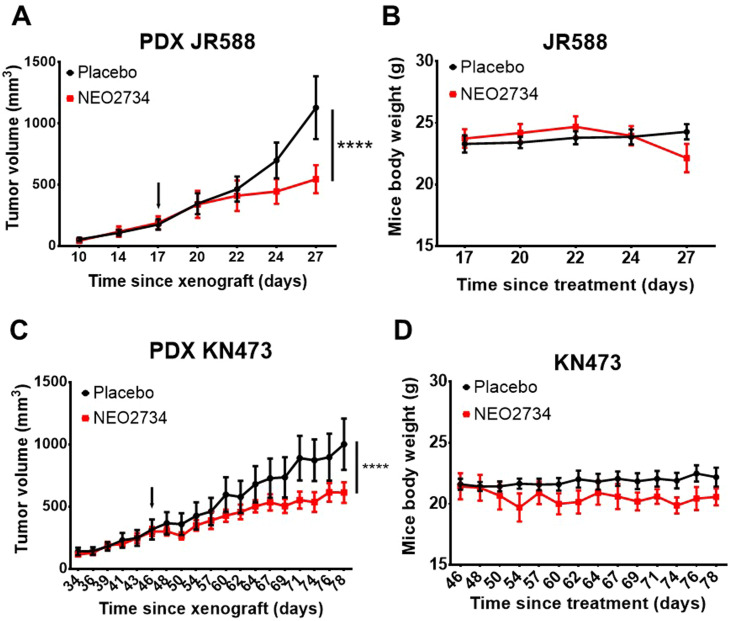
**Efficacy of the NEO2734 inhibitor in vivo in UPS patient-derived xenograft (PDX)**. JR588 **(A and B)** and KN473 **(C and D)** PDX were xenografted in Rag2^-/-^ γc^-/-^ mice (*n* = 8 and *n* = 6 mice per group for JR588 and KN473 respectively) and tumors were treated with vehicle (40 % PEG400) or NEO2734 at 10 mg/kg orally QD. Tumors growth and body weight were monitored (two-way ANOVA and Sidak's multiple comparisons test, arrow = start of treatment).

As NEO2734 was the most potent compound in vitro, we evaluated its efficacy in vivo in two models of UPS patient-derived xenografts (PDXs) (Fig. 3). Tumor growth was reduced in both models. Interestingly, a stronger effect on tumor growth was observed in the JR588 PDX (Fig. 3A) than in the KN473 PDX (Fig. 3C), as observed in in vitro experiments with the corresponding cell lines.

### Identification of NEO2734 resistance gene

**Fig. 4:**
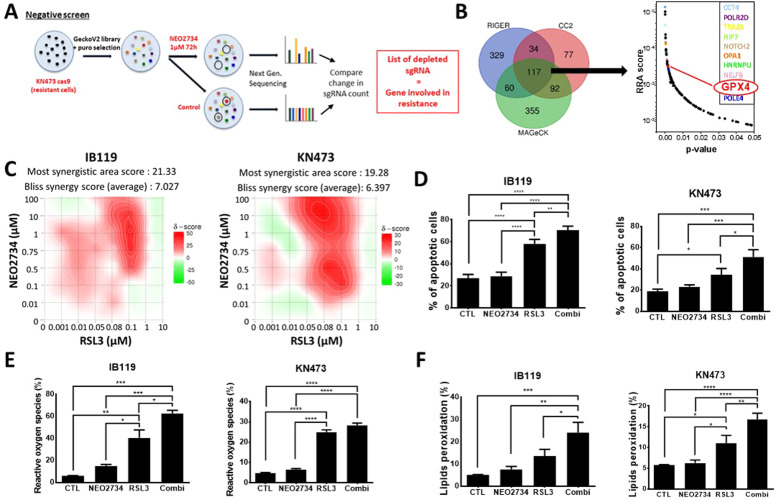
**Synergy between BET/EP300 inhibition and ferroptosis induction. A,** CRISPR knockout screen workflow on KN473 cell lines stably infected with cas9 nuclease and treated with or without NEO2734 at 1 µM for 72 h **B,** Venn diagram of CRISPR screen analyzed by 3 different pipeline (RIGER, CrisprCloud2, MAGeCK) revealed 117 common and significant downregulated genes potentially involved in NEO2734 resistance. Distribution of RRA (Robust Rank Aggregation) score (MAGeCK) and p-values of the 117 common depleted genes. In color, the most interesting genes among the TOP 30 genes. **C,** Synergy maps of RSL3 (GPX4 inhibitor) and NEO2734 combination in IB119 and KN473 cell lines. Regions in red and green colors highlight synergistic and antagonistic dose, respectively. The Bliss method, using the SynergyFinder 3.0 software were used to calculate the synergy score. **D,** Apoptosis in IB119 and KN473 were measured by flow cytometry and Annexin-V/PI staining after 48 h of RSL3 (0.1 µM and 0.06 µM respectively) and/or 1 µM of NEO2734 treatment (*n* = 5, one-way ANOVA and Tukey's multiple comparisons test). **E,** Reactive oxygen species (ROS) measurement in IB119 and KN473 by flow cytometry after DCFDA staining and 24 h of 0.1 µM of RSL3 and/or 1 µM of NEO2734 treatment (*n* = 3, one-way ANOVA and Tukey's multiple comparisons test). **F,** Lipids peroxidation in IB119 and KN473 were measured by flow cytometry after C11-Bodipy staining and 24 h of RSL3 0.1 µM and/or NEO2734 1 µM treatment (*n* = 5, one-way ANOVA and Tukey's multiple comparisons test). *p < 0.05; **p < 0.01; ***p < 0.001; ****p < 0.0001.

Although these results in UPS have been encouraging, lessons from other targeted therapies or BETi in other models indicate that drug resistance is a major problem to BETi responsiveness and cancer treatment in general. As one of our cell lines (KN473) showed a lower sensitivity to NEO2734, we used this line to assess which genes could be involved in this drug resistance and identify a potential combination strategy. We performed a genome-wide pooled lentiviral CRISPR‒Cas9 knockout screen in KN473 cells stably infected with the cas9 nuclease. Following infection with the GeCKO library and selection with puromycin, the cells were divided into two groups: untreated cells versus NEO2734-treated cells. A low dose of 1 µM NEO2734 for 72 hours was deliberately chosen for the KN473 resistant line, as it is not expected to kill cells. Only those sensitized by the guide RNA to this low dose of NEO2734 will be killed. After 72h, genomic DNAs from both group were extracted and sequenced by NGS to identify the guide RNAs depleted after treatment compared with untreated group, i,e., genes whose knockout causes cell death and hypersensitivity to NEO2734 (i.e., genes involved in drug resistance) (Fig. 4A). To select the most important hits, three different pipelines (RIGER, CrisprCloud2, MAGeCK) were used to analyze the CRISPR screen and revealed 117 common and significantly downregulated genes that went from non-essential in the untreated cells to essential in the treated cells and therefore potentially involved in NEO2734 resistance based on the RRA score (Fig. 4B).

Interestingly, several top genes have already been described to be involved in drug resistance. For instance, CCT4 (chaperonin containing TCP-1) knockdown has been shown to enhance the sensitivity of cisplatin in esophageal squamous cell carcinoma [21]. OPA1 (Optic Atrophy Protein 1), a mitochondria-shaping protein, participates in resistance against gefitinib in a lung adenocarcinoma cell line [22] and cisplatin in NSCLC [23].

Among these top genes, GPX4 (glutathione peroxidase 4), which encodes a key enzyme that detoxifies peroxidized lipids and thus inhibits ferroptosis was of particular interest. Indeed, persister cells from breast, melanoma, lung and ovarian cell lines have been shown to acquire a dependency on GPX4 [24]. Furthermore, suppression of GPX4 and SOD2 reversed resistance to EGFR-TKIs in NSCLC [25] and the induction of ferroptosis by impairing STAT3/Nrf2/GPx4 signaling enhances the sensitivity of osteosarcoma cells to cisplatin [26]. Based on this evidence, we reanalyzed the RNAseq data from the sensitive and resistant cell lines following NEO2734 treatment, utilizing the KEGG database, which includes a specific gene set for the ferroptosis pathway. Notably, the ferroptosis pathway was upregulated in both subgroups after treatment (Supplementary Figure 2A and C). This upregulation was significantly more pronounced in the resistant cells, which exhibited a broader panel of upregulated genes associated with this pathway (Supplementary Figure 2B and D). Among these, GPX4 stood out as one of the most significantly upregulated genes in the resistant cell lines (Supplementary Figure 1G), underscoring its potential role in mediating resistance to BET inhibitors.

### Synergy between BET/EP300 inhibition and ferroptosis induction

To assess the role of GPX4 in BET/EP300 resistance, we inhibited GPX4 either by knockout with guide RNA (Suppl Fig 3A and B) or by pharmacological inhibition with RSL3, a specific inhibitor of GPX4 (Suppl Fig 3C). Our findings showed that GPX4 knockout led to a 2.6-fold reduction in the IC50 value of NEO2734 in KN473 cell lines. Using the Bliss synergy model, combining RSL3 and NEO2734 resulted in synergistic antitumor activity in the two resistant cell lines IB119 and KN473 with a most synergistic area (MSA) score of 21.33 and 19.28, respectively (Fig. 4C). It is noteworthy that this MSA is observed at concentrations of NEO2734 between 0.1 and 0.5 µM for KN473 and between 0.75 and 1 µM for IB119. These doses are considerably lower than the IC50 values for NEO2734 in these resistant cell lines. This synergy between GPX4 and BET/P300 inhibition is characterized by an increase in apoptosis (Fig. 4D), reactive oxygen species production (Fig. 4E) and lipid peroxidation (Fig. 4F) in both resistant cell lines treated with the combination of RSL3 and NEO2734. Interestingly, the NEO2734-sensitive cell lines IB106 and JR588 also showed additive or synergistic responses when RSL3 was also applied (MSA scores: 5.53 and 10.18, respectively) (Suppl Fig. 3D), with an increase in ROS for JR588 cells and an increase in apoptosis, ROS and peroxidized lipids for IB106 cells (Suppl Fig. 3E-G). To investigate this synergy in vivo, preliminary safety and efficacy studies were conducted in mice. While RSL3 alone showed a modest reduction in tumor growth, its combination with NEO2734 led to significant toxicity in our experimental setting (intraperitoneal administration of RSL3 at a minimum dose of 25 mg/kg) (data not shown).

## Discussion

Developing precision medicine strategies is of crucial importance to improve the outcome of patients with sarcomas. This requires the identification of complex and unique molecular features that drive these heterogeneous malignancies. Our previous studies revealed two main subclasses of UPS with distinct genomic, immunologic, and proteomic features with potential therapeutic implications [3]. The *immune-enriched* UPS subtype may certainly be the best candidate subtype for immune checkpoint inhibitors. Our present results provide evidence that dual BET/EP300 inhibition has substantial clinical activity in the *immune-low* UPS subtype, which is mainly characterized by enrichment in MYC targets and epithelial mesenchymal transition (EMT) pathways. Interestingly, our CRISPR screen identified GPX4, a major regulator of ferroptosis [27], that could contribute to resistance to dual BET/EP300 inhibition in the clinic. These results are in line with previous studies in epithelial tumors showing synergy between BET inhibitors and ferroptosis induction [28]. In our study, we confirmed the synthetic lethality between dual BET/EP300 inhibition and ferroptosis induction by using both knockout and pharmacological approaches. One limitation of our study is the inability to confirm this synergy in vivo due to safety concerns. While these results are disappointing, our study underscores the potential of ferroptosis induction as a strategy to overcome resistance. Further research is needed to develop safer and more effective RSL3 formulations, potentially through localized delivery methods, to validate this approach in vivo and to gain a deeper understanding of the combination's efficacy and toxicity.

Ferroptosis is a form of cell death driven by iron-dependent phospholipid peroxidation. It has also recently been proven to correlate with cancer therapy resistance and inducing ferroptosis has been demonstrated to reverse drug resistance [29]. Interestingly, Viswanathan et al. showed that mesenchymal cancer cells with high resistance to therapy were dependent on GPX4 for survival [30]. Therefore, owing to their mesenchymal cells of origin and our results, UPS and more broadly sarcomas could be particularly sensitive to this potent combination therapeutic strategy that deserves further investigation in the clinical setting.

## Ethics

This study was approved by the Ethics Committee for Animal Studies at the University of Bordeaux. The animal study was conducted according to the French and European Union guidelines for animal experimentation (RD 1201/05, RD 53/2013 and 86/609/CEE, respectively) and approved by the institutional animal use and care committee under project license APAFiS #8415–2,017,010,211,442,345. The study was also approved by the Institutional Review Board of Institute Bergonié.

## CRediT authorship contribution statement

**Stéphanie Verbeke:** Writing – review & editing, Writing – original draft, Validation, Methodology, Investigation, Formal analysis, Conceptualization. **Aurélien Bourdon:** Formal analysis. **Mathilde Lafon:** Investigation, Data curation. **Vanessa Chaire:** Investigation. **Bertolo Frederic:** Investigation. **Amina Naït Eldjoudi:** Investigation. **Marie-Alix Derieppe:** Investigation. **Francis Giles:** Resources. **Antoine Italiano:** Writing – review & editing, Writing – original draft, Validation, Supervision, Resources, Project administration, Methodology, Investigation, Funding acquisition, Formal analysis, Data curation, Conceptualization.

## Declaration of competing interest

The authors declare that they have no known competing financial interests or personal relationships that could have appeared to influence the work reported in this paper.

## Appendix Supplementary materials

Image, application 1
