# Supplementary material for: Dual inhibition of BET and EP300 has antitumor activity in undifferentiated pleomorphic sarcomas and synergizes with ferroptosis induction
Source: Transl Oncol. 2024 Dec 15;52:102236. doi: 10.1016/j.tranon.2024.102236 (PMC11713734; doi:10.1016/j.tranon.2024.102236)
Supplement: Supplementary file 1 [file mmc1.docx]

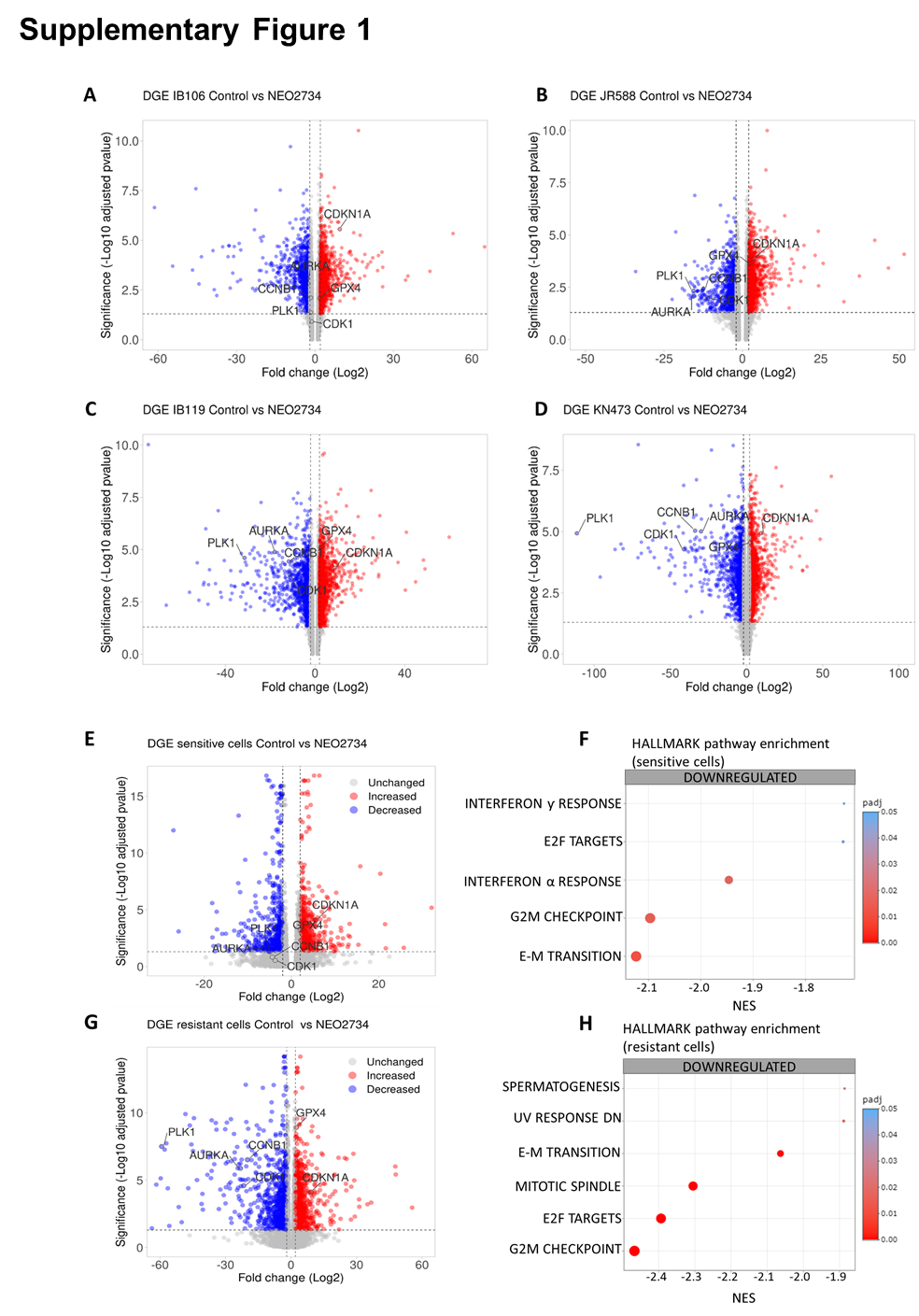


**Supplementary Fig. 1: Transcriptomic analysis of dual BET/EP300 inhibitor effect**

Volcano plot of differential gene expression (DGE) analysis after RNA sequencing of IB106 **(A)**, JR588 **(B)**, IB119 **(C)**, KN473 **(D)** cell lines treated or not with NEO2734 for 72 h at their respective IC50 (25 µM for IB119 and KN473) in triplicate. Genes in blue and genes in red were significantly down- or up-regulated respectively after treatment. The threshold was –log10 adjusted *p* < 0.05 and log2 fold change > 2. **E and G:** Volcano plot of differential genes expression analysis after NEO2734 treatment in sensitive (**E**) and resistant (**G**) cell lines respectively**. F and H:** HALLMARK pathway enrichment analysis after NEO2734 treatment in sensitive (**F**) and resistant **(H)** cell lines.


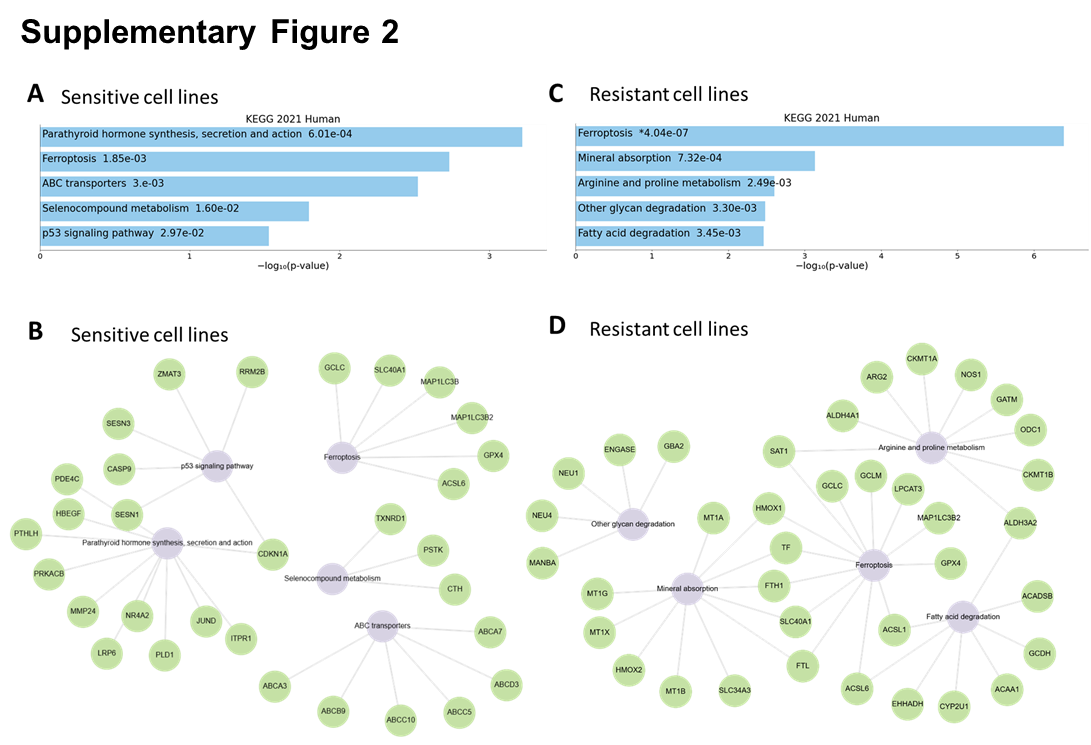


**Supplementary Fig. 2: Upregulated pathways after NEO2734 treatment (KEGG database)**

**A and C:** KEGG pathway enrichment analysis after NEO2734 treatment in sensitive (**B**) and resistant (**D**) cell lines. Colored bars correspond to terms with significant p-values (<0.05). An asterisk (*) next to a p-value indicates the term also has a significant adjusted p-value (<0.05). **B and D:** Network view of the interconnections between genes and their associated regulated pathways using EnrichR-KG.


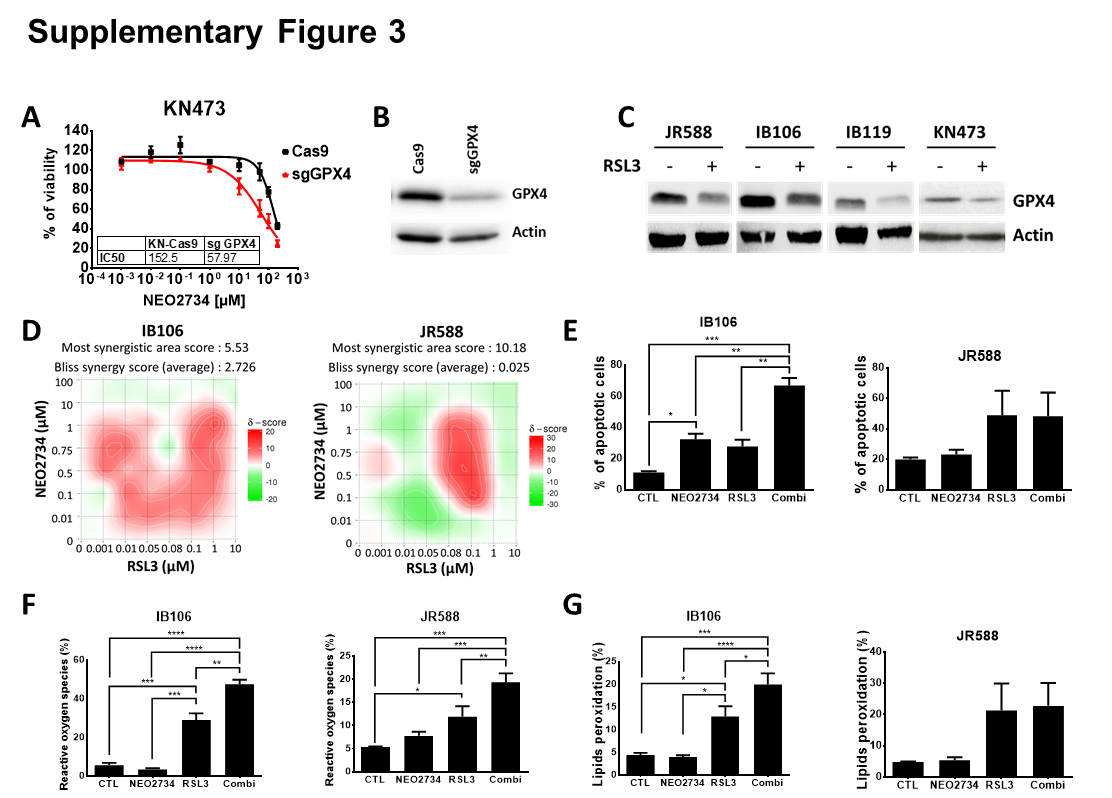


**Supplementary Fig. 3: Synergy between BET inhibitor NEO2734 and GPX4 inhibition**.

**A,** Assessment of cell viability by MTT assay of KN473 cell lines stably infected with cas9 nuclease alone or with guide RNA targeted GPX4 after NEO2734 treatment. Cells were treated with a range of increasing concentrations of NEO2734 for 72 h and IC50 were calculated with GraphPad Prism software (*n* = 5). **B,** Western blot of GPX4 on KN473 cell line stably infected with cas9 nuclease alone or with guide RNA targeted GPX4. Actin was used as loading control. **C,** Western blot of GPX4 on 4 UPS cell lines treated or not for 24 h with RSL3, a GPX4 pharmacological inhibitor (JR588/IB119/KN473: 0.1 µM; IB106: 0.8 µM). Actin was used as loading control. **D,** Synergy maps of RSL3 and NEO2734 combination in IB106 and JR588 cell lines. Regions in red and green colors highlight synergistic and antagonistic dose, respectively. The Biss method, using the SynergyFinder 3.0 software were used to calculate the synergy score. **E,** Apoptosis in IB106 and JR588 were measured by flow cytometry and Annexin-V/PI staining after 48 h of RSL3 (0.8 µM and 0.09 µM respectively) and/or NEO2734 treatment (1 µM and 0.2 µM respectively) (*n* = 3, one-way ANOVA and Tukey’s multiple comparisons test). **F,** Reactive oxygen species (ROS) measurement in IB106 and JR588 by flow cytometry after DCFDA staining and 24 h of RSL3 (0.8 µM and 0.06 µM respectively) and/or NEO2734 treatment (1 µM and 0.2 µM respectively) (*n* = 3, one-way ANOVA and Tukey’s multiple comparisons test). **G,** Lipids peroxidation in IB106 and JR588 were measured by flow cytometry after C11-Bodipy staining and 24 h of RSL3 (0.5 µM and 0.09 µM respectively) and/or NEO2734 treatment (1 µM and 0.2 µM respectively) (*n* = 5, one-way ANOVA and Tukey’s multiple comparisons test). **p* < 0.05; ***p* < 0.01; ****p* < 0.001; *****p* < 0.0001
